# A Piglet Model for Detection of Hypoxic-Ischemic Brain Injury with Magnetic Resonance Imaging

**DOI:** 10.1080/02841850802334224

**Published:** 2008-10-16

**Authors:** B. H. Munkeby, C. De Lange, K. E. Emblem, A. Bjørnerud, G. A. B. Kro, J. Andresen, E. H. Winther-Larssen, E. M. Løberg, J. K. Hald

**Affiliations:** Department of Paediatric Research, Institute for Surgical Research, Department of Radiology, Department of Medical Physics, Intervention Center, and Department of Gynecology and Obstetrics, Rikshospitalet University Hospital, Oslo, Norway, Institute of Physics, University of Oslo, Oslo, Norway and Department of Pathology, Ullevål University Hospital, Oslo, Norway

**Keywords:** Animal investigations, brain/brainstem, CNS, MR diffusion/perfusion, MR imaging, MR spectroscopy

## Abstract

Munkeby BH, de Lange C, Emblem KE, Bjørnerud A, Kro GAB, Andresen J, Winther-Larssen EH, Løberg EM, Hald JK. A piglet model for detection of hypoxic-ischemic brain injury with magnetic resonance imaging. Acta Radiol 2008;49:1049–1057.

**Background:**

Early detection of hypoxic-ischemic (HI) injury in the asphyxic newborn is important because present prognostic factors are inadequate. Furthermore, therapeutic interventions may have additional benefit if initiated in time.

**Purpose:**

To assess whether the use of a combined protocol including conventional magnetic resonance imaging (MRI), diffusion-weighted imaging (DWI), diffusion tensor imaging (DTI), and proton MR spectroscopy (MRS) could detect pathological findings in a piglet model 7 hours after HI.

**Material and Methods:**

Ten piglets were submitted to HI for 30 min followed by reoxygenation with 21% O_2_ for 7 hours. MRI at 1.5T was done prior to and 7 hours after the HI. Single-voxel proton MRS was performed, and apparent diffusion coefficient (ADC) and fractional anisotropy (FA) were measured in the basal ganglia. MRS identified N-acetylaspartate (NAA), choline (Cho), creatine (Cr), and lactate (Lac). Histology and microtubule-associated protein 2 (MAP-2) staining was performed in the basal ganglia at the end of the experiment.

**Results:**

Compared to baseline, ADC, NAA/Cho, and NAA/Cr were significantly reduced after 7 hours (*P* < 0.001, *P* = 00.01, and *P* = 00.05, respectively) and FA values were increased (*P* <0.025). The ratios of Lac/Cho and Lac/NAA were significantly higher after 7 hours compared to baseline (*P* <0.001). Presence of necrosis correlated well with reduced ADC (R_S_ = 0.91) and presence of Lac (R_S_ = 0.80). Histology and MAP-2 staining showed more than 90% necrosis in eight piglets, 60% in one piglet, and no necrosis in one piglet.

**Conclusion:**

Diffusion MRI and proton MRS can detect HI injury in the piglet brain 7 hours after hypoxia. DWI and MRS can be used to give useful prognostic information. This piglet model may potentially be used to mimic clinical situations and is suitable for further research investigating HI injury.

Some 5.2% of infants out of 130 million annual births suffer from birth asphyxia. Of these, 25% die and 25% develop some kind of sequelae (1). Clinical characteristics are not sufficient to determine etiology or prognosis in term infants with encephalopathy, particularly in the absence of a clear history of asphyxia (2).

It is well known that diffusion magnetic resonance imaging (MRI) and proton MR spectroscopy (MRS) are valid methods for evaluating early alterations in asphyxia, both in human babies and animals (3). Several studies have shown that MRI is the modality of choice in studies of brain injury in term neonates (4) and in newborn piglets (5, 6). Following perinatal hypoxic-ischemic (HI) injury, proton MRS may be a valuable tool in providing information on timing and pattern of acute brain metabolite changes. Increased levels of lactate have been detected in neonates with subsequent poor neurodevelopmental outcome and have been characterized as a prognostic factor (7). MR-based diffusion-weighted imaging (DWI) is sensitive to changes in water diffusion, in both magnitude and direction. Many studies have shown that the apparent diffusion coefficient (ADC), as measured by DWI, is reduced in the hyperacute phase following an HI event due to influx of water from the extra- to the intracellular space (8). A previous study has shown that ADC reduction during HI in a piglet model is reversible after 30 min (9).

In the setting of acute stroke, fractional anisotropy (FA) correlates with time of stroke onset (10). Clinical studies conclude that anisotropy measurements are promising for the early detection of impaired brain development in neonates (11). Yang et al. described three temporally related different phases in the relationship between FA and ADC (12). There are only a limited number of studies which have investigated the effect of hypoxia-ischemia on diffusion anisotropy in the acute phase. In this study, the measurements were performed at baseline and 7 hours after HI. A 7-hour observation period gives us the opportunity to investigate changes in the early phases of secondary energy failure, which occurs from 6 to 48 hours after the primary insult (13, 14).

The purpose of our study was to describe how MRS and DWI can be added to a piglet model that may potentially be used to mimic clinical situations. Thus, our hypothesis is that these MR techniques are suitable for early assessment of acute tissue changes in the piglet brain after HI damage.

## Material and Methods

The experimental protocol was approved by the hospital’s ethics committee for animal studies under the surveillance of the National Animal Research Authority, and performed by certified category C researchers of the Federation of European Laboratory Animal Science Associations.

### Anesthesia and surgical preparation

Ten newborn, anesthetized, and tracheotomized Noroc (LY × LD) pigs born at term (12–36 hours of age) were included in the study, inclusion criteria being hemoglobin values > 5 g/dl, and good general condition. The animals had a mean (SD) weight of 3.03 (±0.16) kg, hemoglobin 7.2 (±1.7) g/dl, and hypoxic time of 30 min. There were five female and five male piglets.

Anesthesia was induced by sevoflurane 5% (Sevorane; Abbott, Oslo, Norway); an ear vein was cannulated, sevoflurane was disconnected, and the piglets were given pentobarbital sodium 20 mg/kg (Abbott, Oslo, Norway) and fentanyl 50 μg/kg (Alpharma, Oslo, Norway) intravenously as a bolus injection. Anesthesia was maintained by a continuous infusion of fentanyl 50 μg/kg/hour and midazolam 0.25 mg/kg/hour (Alpharma, Oslo, Norway) by an IVAC P2000 Syringe Pump (Alaris Medical Systems, Cardiff, UK). When necessary, a bolus of fentanyl 10 mg or midazolam 1 mg was added. A continuous intravenous infusion of Salidex 10 ml/kg/hour (Braun, Oslo, Norway) was given throughout the experiment outside the MR suite. Tracheostomy was performed, and a pressure-controlled ventilator (Babylog 8000 +; Drägerwerk, Lübeck, Germany) ventilated the piglets at a rate of 30 breaths/min. Normoventilation (arterial carbon dioxide tension [PaCO_2_] 4.5–6.0 kPa, saturation ≥90%) and a tidal volume of 6–15 ml/kg were achieved by adjusting the peak inspiratory pressure or ventilatory rate, and, if needed, the fraction of inspired oxygen (FiO_2_). Inspiratory time of 0.4 s and positive end-expiratory pressure of 4 cm H_2_O were kept constant throughout the experiment. Inspired fraction of O_2_ and end-tidal CO_2_ were monitored continuously (Datex Normocap Oxy; Datex, Helsinki, Finland). After stabilization, the piglets were transported from the operation theater to the MRI suite in an incubator with an inner temperature of 38°C. Anesthesia in the MR suite was maintained with a continuous inhalation of isoflurane (Abbot Scandinavia AB, Kista, Sweden) at 1–1.5% minimum alveolar concentration (MAC) and a mixture of N_2_O (30%) and O_2_ (70%) and an hourly bolus injection of fentanyl 50 μg/kg. During the experiment, the animals were monitored by an Invivo 3150 (OMNI-Trak, 3150 MRI-Monitor, Orlando, Fla., USA), measuring heart rate, peripheral oxygen saturation, end-tidal CO_2_, and invasive blood pressure.

The left femoral artery and vein were cannulated with polyethylene catheters (Portex PE-50, inner diameter 0.58 mm; Portex Ltd., Hythe, Kent, UK). The common carotid arteries on both sides were exposed through a small incision in the neck at the level of the fourth cervical vertebra.

Rectal temperature was maintained between 38 and 40°C with a heating blanket and a radiant heating lamp. At the end of the experiment, the piglets were given an overdose of 150 mg/kg pento-barbital intravenously.

### Experimental model

After 1 hour of stabilization, the piglets were subjected to HI for 30 min, 8% O_2_ in N_2_ (AGA, Oslo, Norway), and, simultaneously, bilateral carotid clamping of the common carotid arteries, followed by 7 hours’ reoxygenation with ambient air (21% O_2_) and reperfusion (9). The clamping of the common carotid arteries was verified by two-dimensional (2D) ultrasonography with color and spectral Doppler.

### Magnetic resonance imaging

MRI was performed at 1.5T (Siemens Sonata; Siemens, Erlangen, Germany). The anesthetized piglets were imaged with a quadrature extremity coil. MRI was performed prior to hypoxia (15) and 7 hours after the hypoxic event. The MRI protocol included: axial and coronal 2D turbo spin-echo (TSE) T2-weighted images (repetition/echo time [TR/TE] 4000/90 ms, slice thickness 2 mm, 336 × 512 matrix, field of view [FOV] 175 × 200 mm^2^), coronal 2D fluid-attenuated inversion recovery (FLAIR) images (TR/TE/inversion time [TI] 9000/ 108/2500 ms, slice thickness 3 mm, 224 × 256 matrix, FOV 166 × 190 mm^2^), three-directional echoplanar SE diffusion-weighted images (TR/TE 2900/84 ms, slice thickness 3 mm, 128 × 128 matrix, FOV 200)200 mm^2^, *b* values 0, 500, and 1000 s/mm^2^), and 12-directional echo-planar SE diffusion tensor images (TE/TR 80/3000 ms, *b* value 750 s/mm^2^, 112 × 128 matrix, FOV 175 × 200 mm^2^). Single-voxel proton MRS (PRESS, TE/TR 270/1600 ms, voxel dimension 10 × 10 × 10 mm) was obtained from the basal ganglia, identifying N-acetylaspartate (NAA), choline (Cho), creatine (CR), and lactate (Lac).

### Image analysis

**Fig. 1:**
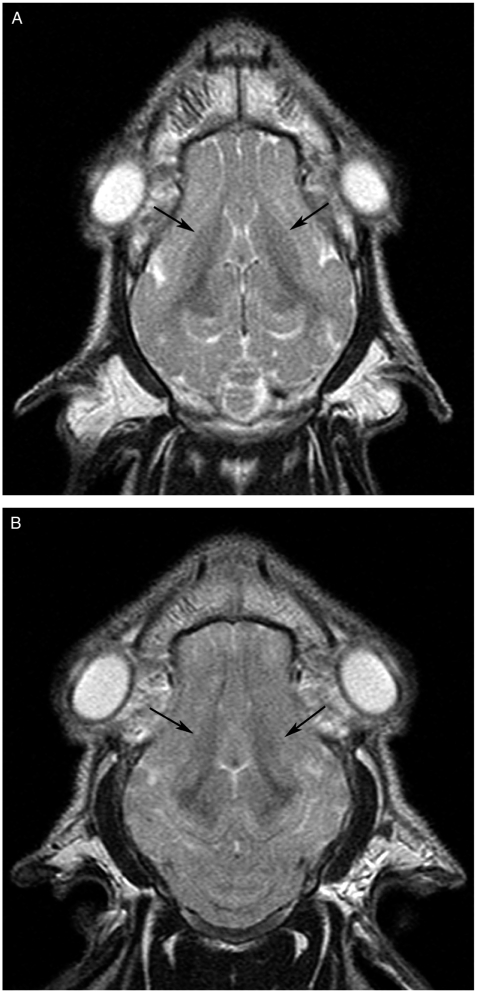
Morphological changes visualized by T2 in the basal ganglia. Axial T2-weighted spin-echo images (TR/TE 4000/90 ms) of subject 8 prior to HI (A) and 7 hours after HI (B). The most prominent morphological change seen on the MR images after 7 hours was a slight relative increase in signal intensity in the basal ganglia with effacement of borders post-HI (arrows).

Morphological changes on T2- and FLAIR-weighted sequences were evaluated by consensus by two experienced radiologists. The regions of the basal ganglia, cerebral cortex, subcortical white matter, brainstem, and cerebellum were analyzed for low- or high-signal-intensity lesions and absence or presence of edema (defined as sulcal and ventricular effacement), before and after the HI insult (Fig. 1).

Postprocessing was performed on a standard Siemens Sonata workstation (doctor’s console). From the MRS spectra, four metabolites were measured: Cho (peak 3.19–3.29 ppm), Cr (peak at 2.99–3.10 ppm), NAA (peak at 1.98–2.08 ppm), and lactate doublet (peak at 1.32–1.40 ppm). Ratios of NAA/Cho and NAA/Cr were calculated using integral values, and the presence or absence of a Lac peak was recorded.

**Fig. 2:**
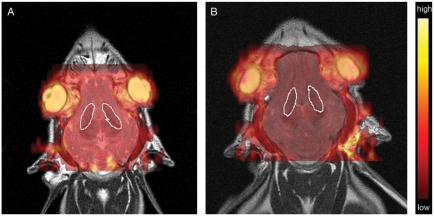
Apparent diffusion coefficient (ADC) maps overlaid on axial T2-weighted spin-echo images (TR/TE 4000/90 ms) of subject 8 prior to HI (A) and 7 hours after HI (B). The regions of interest (ROI) marked with an arrow illustrate the area used for measuring the ADC values in the basal ganglia.

Apparent diffusion coefficient (ADC) maps and fractional anisotropy (FA) maps were created from the diffusion-weighted images and the diffusion tensor images, respectively, using previously described methods (14). ADC and FA maps were coregistered with the conventional MR images, and regions of interest (ROIs) were drawn around the basal ganglia in each hemisphere, by the same radiologists (Fig. 2). Mean ADC and FA values with standard deviations for the piglets as a group at both time points were recorded (approximately *n* = 8500 ADC pixel values in each cohort). All diffusion-related analyses and image coregistrations were calculated using dedicated software (nordicICE; NordicImagingLab, Bergen, Norway).

### Pathological examination

#### Fixation and staining

Tissue blocks (0.5 cm thick) from the basal ganglia were embedded in paraffin, sliced in 4-μm-thick sections, and stained with hematoxylin and eosin (H&E).

#### Immunohistochemistry

Formalin-fixed paraffinembedded sections from the basal ganglia were deparaffinized, rehydrated, and demasked in a microwave oven for 15 min in Tris/EDTA at pH 9.1. Monoclonal anti-MAP-2 a & b, titer 1:300 (Chemicon International, Temecula, Calif., USA) was used as a primary antibody. The antigen–antibody reaction was visualized with the DAKO EnVision horseradish peroxidase system (DAKO Cytomation Norden A/S, Glostrup, Denmark) using 3.3’-diaminobenzidin as the chromogen.

#### Evaluation

The samples were evaluated by a pathologist. Due to the short time of survival between the hypoxic-ischemic event and the sacrifice, the morphological changes in the basal ganglia were subtle. Areas with vacuolated neutrophils, shrunken neurons with pyknotic nuclei, and scattered eosinophilic neurons were defined as early necrosis. MAP-2, which is a sensitive marker for neuronal ischemic injury (16), was used to confirmthe areas of damage. Necrotic areas showed loss of MAP-2 staining. The damage was reported as <10%, 10–30%, 30–60%, 60–90%, or >90%.

### Statistical analysis

For each piglet, the ADC and FA pixel values from the ROIs in the basal ganglia at baseline were compared to those after 7 hours using a Student *t* test with a significance level of *P* = 0.05 (approximately *n* = 500 pixel values in each cohort). The ADC and FA values which best differentiated between baseline and 7 hours after HI were obtained using binary logistic regression. The difference between mean ADC values at baseline and after 7 hours for the piglets as a group was evaluated using Mann-Whitney tests with a significance level of *P* = 0.05. The same procedure (Mann-Whitney, *P* = 0.05) was calculated for mean FA values and for the ratios of NAA/Cho, NAA/Cr, Lac/Cho, and Lac/NAA. For each piglet, a significant difference between values at baseline and after 7 hours was thought to represent an injury. The correlation between the results from histology and MR was evaluated for the ADC values, the FA values, and the presence of lactate (yes/no) using the Spearman rank-correlation coefficient (*R*_s_). Statistical analysis was performed using SPSS version 13 (Apache Software Foundation, Chicago, Ill., USA).

## Results

### MRI and MRS

The baseline T2-weighted TSE and FLAIR images verified that there were no previous cerebral injuries that could have influenced the results. Morphological changes consisting of light global edema were present in four of the 10 piglets on the MRI scans performed 7 hours after the HI insult. High relative signal intensity in the regions of the basal ganglia on T2-weighted or FLAIR images was present in two out of 10 (Fig. 1) and slight effacement of the basal ganglia contours in five out of 10 brains after HI.

**Fig. 3:**
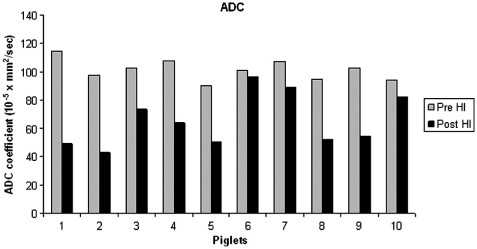
Apparent diffusion coefficient (ADC) values in the basal ganglia for the 10 piglets prior to HI (gray bars) and after 7 hours (black bars). Compared to baseline MRI, the ADC values in the basal ganglia were significantly lower after 7 hours (Mann-Whitney: *P*<0.001).

All piglets had significantly lower ADC values in the basal ganglia after 7 hours compared to the baseline ADC values (*P*<0.001). Mean ADC values in the basal ganglia for the piglets as a group at baseline and after 7 hours were 101.37±12.44×10^−5^mm^2^/s and 58.12±16.14)×10^−5^mm^2^/s, respectively (Fig. 3). The optimal cut-off ADC value to differentiate baseline ADC values and values after 7 hours was 82.96×10^−5^mm^2^/s. There was a strong correlation between reduced ADC value after 7 hours and ischemic injury as determined by histology (*R*_s_=0.905, *P*=0.01).

The mean FA values in the basal ganglia were significantly higher after 7 hours compared to baseline (0.499±0.082 vs. 0.474±0.029, *P*<0.025). However, three piglets (piglets 1, 4, and 5) had reduced FA values (*P*<0.001), whereas the remaining seven piglets had significantly higher FA values after 7 hours compared to the baseline (*P*<0.001). Thus, correlation statistics were not calculated for the FA index.

**Fig. 4:**
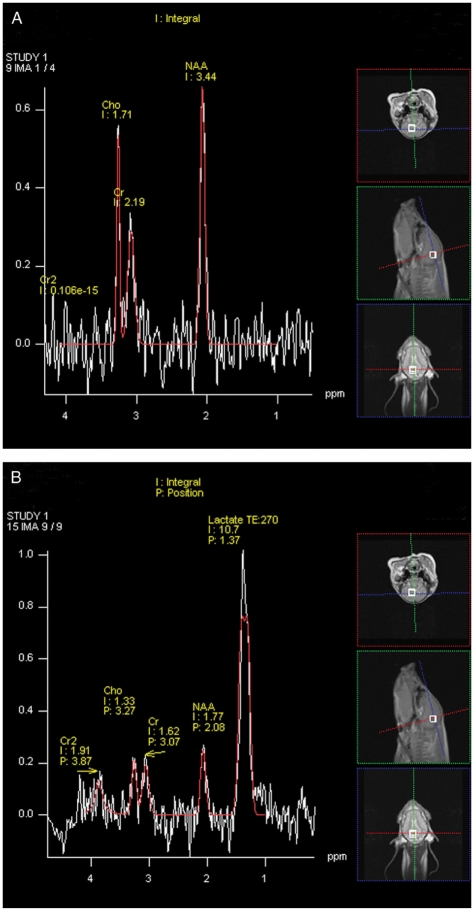
Proton MR spectroscopy spectra (single voxel, PRESS) of subject 8 prior to HI (A) and after 7 hours (B). Integral (I) and position (P) values of choline (Cho), creatine (Cr), N-acetylaspartate (NAA), and lactate (Lac) were recorded in the basal ganglia.

**Fig. 5:**
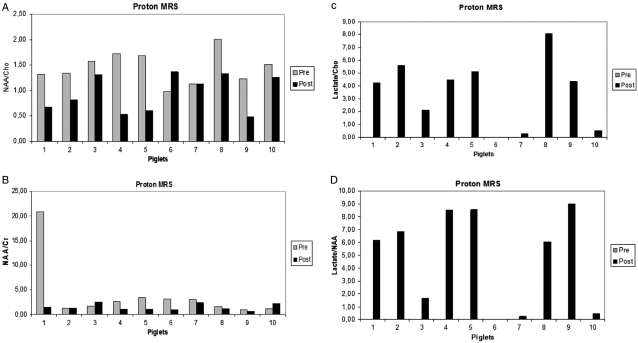
Proton MR spectroscopy integral values prior to HI (gray bars) and 7 hours after HI (black bars). The ratios of NAA/Cho (A) and NAA/Cr (B) were significantly lower after 7 hours (*P*=0.011 and *P*=0.049, respectively), whereas the ratios of Lac/Cho (C) and Lac/NAA (D) were significantly higher after 7 hours (*P*<0.001).

With respect to proton MRS (Fig. 4), the ratios of NAA/Cho and NAA/Cr were significantly lower after 7 hours compared to baseline (*P*=0.011 and *P*=0.049, respectively) (Fig. 5A and B). The ratios of Lac/Cho and Lac/NAA were significantly higher after 7 hours compared to baseline (*P*<0.001) (Fig. 5C and D). One of the 10 piglets (piglet 6) had no increased lactate after 7 hours, corresponding to the piglet with the lowest relative change in ADC. There was a strong correlation between the presence of lactate and the presence of necrosis as determined by histology (*R*_s_=0.798, *P*=0.01).

### Histology and MAP-2

**Fig. 6:**
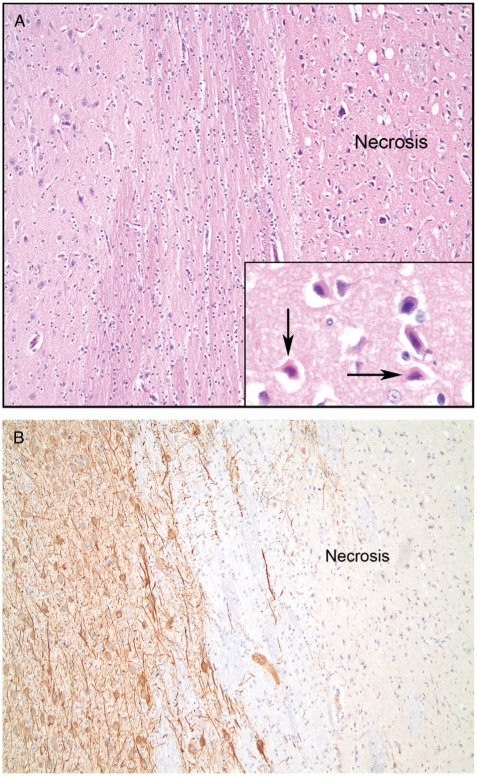
A. Early necrosis with shrunken and eosinophilic neurons, H&E, 10x. Inset shows eosinophilic neurons (arrows), 40x. B. Early necrosis with loss of MAP-2 staining, 10x.

There was a good correlation between the morphologic changes in the HE-stained sections and loss of MAP-2 staining, as shown in Fig. 6. In eight of 10 piglets, we found widespread necrotic areas in the basal ganglia, involving 90–100% of the tissue, and one piglet presented damage to 60% of the tissue, 7 hours after hypoxia. One piglet (piglet 4) showed no histological signs of necrosis.

## Discussion

The availability of a well-controlled piglet model for investigating neonatal injury due to HI is of great importance (17), and in this study we demonstrate correlation between MR findings and histopatho-logical changes in one such model, with results similar to those of *a* recent retrospective study of human newborns with HI injury (18). In this study, proton spectra were localized to the basal ganglia because previous studies using MRI have identified this area as vulnerable to hypoxia-ischemia injury in neonates (19). Spectra from this region are also less susceptible to interference arising from extracranial fat tissue (20).

Reduced ADC and the presence of lactate were strongly correlated to tissue damage. Whereas all piglets showed signs of cerebral pathology in the basal ganglia on diffusion-weighted MRI, MRS, or histology after the HI insult, one of the 10 piglets had no increased lactate and another had no histological changes. The ratios of Lac/Cho and Lac/NAA were significantly higher after 7 hours compared to baseline. Presence of a lactate peak a few hours after birth may be due to residual lactate from the primary insult and may subsequently resolve. Therefore, the presence of lactate in the first hours after an HI injury may not carry the same adverse prognosis as later elevation seen during secondary energy failure (21).

Our increased ratios of Lac/Cho and Lac/NAA, and decreased ratios of NAA/Cho and NAA/Cr 7 hours after HI are consistent with previous data (2). These findings correlate with poor prognosis in clinical studies (22), and confirm the important role of proton MRS in assigning an early prognosis of neonates with neonatal encephalopathy (23). Morphological changes, as seen on T2-weighted TSE and FLAIR images, were variable and only present in half of the piglet brains, 7 hours after HI. It is well known that acute ischemic cerebral changes cannot be reliably identified with these sequences (24).

The age of the animals is very important when studying HI. It has been shown that a difference of just 1 day in rats has a profound impact on the severity of the injury as assessed by spectroscopy and histology (25). Grate et al. showed that piglets and humans are comparable at birth regarding brain maturation (26). We do not know the exact age in hours of each piglet. The piglets were brought to us on the day of the experiment, and they were close to but less than 36 hours old. Therefore, age variation in the present study may not have affected the results.

Niblock et al. (27) showed that 5-HT (serotonergic systems) receptor binding profiles in selected nuclei in the two species suggest that the equivalent postnatal ages for 5-HT development in piglets and human infants are 4 days and 1 month, respectively.

The almost instant reduction in tissue water diffusion following cerebral ischemia is well documented and was demonstrated with diffusion-weighted MRI in cats already in 1990 (28). The mechanism behind the diffusion reduction during ischemia is still not fully understood, but is thought to be mainly caused by cytotoxic edema which results from the breakdown of the cellular membrane Na/K pump system (29). DWI has recently been shown to be a very sensitive method to diagnose severe neonatal HI. In a recent study in five neonates with severe HI, ADC was shown to be significantly reduced in the cerebrum relative to the unaffected cerebellum (30). The mean ADC values obtained in this study at baseline are comparable with previously reported values, with a baseline ADC value of 101±7×10^−5^mm^2^/s in piglets, with a reduction to 69916)10^−5^mm^2^/s during HI (9), whereas a study in control infants reported baseline ADC values in the basal ganglia in the range of 103–127×10^−5^mm^2^/s (31). A previous study also observed a gradual increase in ADC toward baseline in the first 2 hours after HI (9). This may represent transient normalization. In the current study, the persistent reduction in ADC 7 hours after HI is consistent with the histological confirmation of irreversible cell damage. This was also confirmed by the presence of lactate in the basal ganglia at the time of post-HI imaging.

For the piglets as a group, the FA values in the basal ganglia increased 7 hours after HI compared to baseline. Our FA and ADC data correlate with previous studies (32). As cytotoxic edema develops, there is a shift of water from the extracellular to the intracellular space, but the cell membrane remains intact and there is no overall increase in tissue water. This would explain elevated FA, reduced ADC, and normal T2 (33).

In our study, three piglets had significantly decreased FA values 7 hours after HI. Thus, our results suggest that detecting ischemic injury 7 hours after HI using FA values is less conclusive than using ADC values.

The use of MAP-2 immunohistochemistry made it possible to find areas with loss of MAP-2 staining as an indicator of damage, and it was possible to compare the HE-stained areas and the areas with loss of MAP-2 staining to confirm the necrosis seen with light microscopy. Despite the short time of observation, there were widespread necrotic areas in the basal ganglia. The distribution of brain damage has been demonstrated earlier (34). Martin et al. (35) showed that necrotic damage to the striatum occurs relatively soon in newborn piglets subjected to hypoxia, and Thoresen et al. (36) also demonstrated the distribution of hypoxic-ischemic brain damage in the newborn piglet.

Animal models will always be an approximation to the clinical situation. We used a piglet model because the anatomy and physiology are similar to humans, and the cerebral maturation and myelinization of the newborn pig is comparable to the human neonate (37). A weakness of the current study is the fact that the animals were 12–36 hours old, and therefore to some extent adapted to extrauterine life. Another limitation is that the current model does not allow long-term follow-up studies in its current form. Histological findings in this model do, however, confirm considerable brain damage as early as 150 min after the insult (38). Vannucci et al. observed linear relationships between histological tissue injury and high-energy phosphate concentrations at 6 to 18 hours after HI and with much greater significance at 24 to 48 hours (39).

A study with longitudinal investigation of MRI, DWI, DTI, and proton MRS with the use of higher field strengths is currently being conducted by our group. Whether our findings can be applied to detect HI injury in asphyxiated newborn infants should be settled through clinical trials.

It is recommended to collect data that enable calculation of brain metabolite concentrations (expressed as milli-mole per kilogram wet weight of brain tissue) (40). However, only a few studies have measured absolute metabolite concentrations (21). In the current study, it was not technically feasible to perform quantitative spectroscopy.

The combination of neurological assessment and MRS is thought to provide the most reliable prediction of neuron developmental outcome at 1 year of age (21). The fact that our experimental model requires general anesthesia, and that we do not observe the animals in a wake state, makes neurobehavioral testing impossible. Longer observation time and neurobehavioral testing would be desirable for further information.

In conclusion, our data correlate with findings in clinical studies, indicating that this piglet model has the potential to mimic clinical situations. The model is suitable for further research investigating HI injuries in which DWI and MRS can be used to study the effects of early neuroprotective treatment.
